# Comparing 5-Year Survival Rates Before and After Re-stratification of Stage I–III Right-Sided Colon Cancer Patients by Establishing the Presence/Absence of Occult Tumor Cells and Lymph Node Metastases in the Different Levels of Surgical Dissection

**DOI:** 10.1007/s11605-022-05434-6

**Published:** 2022-08-29

**Authors:** G. S. Banipal, B. V. Stimec, S. N. Andersen, A. E. Faerden, B. Edwin, J. Baral, J. M. Nesgaard, J. Šaltytė Benth, D. Ignjatovic, For the R. C. C. study group, Tom Oresland, Arne O. Bakka, Yngve Thorsen, Anne Negaard, Russel Jacobsen, Kari Mette Langerød von Brandis, Tania Hansen, Pål Suhrke, Javier Luzon, Barış Sevinç, Bjarte Tidemann Andersen, Roberto Bergamaschi, Frieder Pullig, Ulrich Schneider, Marcos Gomez Ruiz, Erik Kjaestad, Vahid Bemanian, Anne Pernille H. Dyrbekk, Vladimir Zivanovic, Johannes Kurt Schultz, Knut Magne Augestad, Hanne Marie Hamre

**Affiliations:** 1 Department of Digestive Surgery, Akershus University Hospital HF, Postboks 1000, 1478 Lorenskog, Norway; 2 Institute of Clinical Medicine, Faculty of Medicine, University of Oslo, Oslo, Norway; 3 Anatomy Sector, Teaching Unit, Faculty of Medicine, University of Geneva, Geneva, Switzerland; 4 Department of Pathology, Akershus University Hospital, Lorenskog, Norway; 5 Interventional Centre and Dep. of HPB Surgery, Rikshospitalet, Oslo University Hospital, Oslo, Norway; 6 Department of Colorectal Surgery, Klinikum Karlsruhe, Teaching Hospital University Freiburg/Breisgau, Freiburg, Germany; 7 Department of Digestive Surgery, Vestfold Hospital Trust, Tonsberg, Norway; 8 Health Services Research Unit, Akershus University Hospital, Lorenskog, Norway

**Keywords:** Colon cancer, Occult tumor cells, Stages I–III, D3 right colectomy, Extended D3 mesenterectomy

## Abstract

**Background:**

To establish the impact of re-stratification on the outcomes of patients (stage I–III right-sided colon cancer) based on the presence/absence of occult tumor cells (OTC) and/or metastatic lymph nodes in the different levels of surgical dissection.

**Methods:**

Consecutive patients were drawn from a multicenter prospective trial. After surgery, the surgical specimen was divided into the D1/D2 and D3 volumes before being further analyzed separately. All lymph nodes were examined with cytokeratin CAM 5.2 immunohistochemically. Lymph nodes containing metastases and OTC (micrometastases; isolated tumor cells) were identified. Re-stratification was as follows: RS1, stages I/II, no OTC in D1/D2 and D3 volumes; RS2, stages I/II, OTC in D1/D2 and/or D3; RS3, stage III, lymph node metastases in D1/D2, with/without OTC in D3; RS4, stage III, lymph node metastases in D3, with/without OTC in D3.

**Results:**

Eighty-seven patients (39 men, 68.4 + 9.9 years) were included. The standard stratified (SS) group contained the following: stages I/II (SS1) 57 patients; stage III (SS2) 30 patients. Re-stratified (RS) contained RS1 (38), RS2 (19), RS3 (24), and RS4 (6) patients. Lymph node ratio (OTC) RS2: 0.157 D1/D2; 0.035 D3 and 0.092 complete specimens. Lymph node ratio RS3: 0.113 D1/D2; complete specimen 0.056. Overall survival and disease-free survival were *p* = 0.875 and *p* = 0.049 for SS and *p* = 0.144 and *p* = 0.001 for RS groups, respectively.

**Conclusion:**

This re-stratification identifies a patient group with poor prognosis (RS4). Removing this group from SS2 eliminates all the differences in survival between RS2 and RS3 groups. The level of dissection of the affected nodes may have an impact on survival.

**Clinical Trial:**

“Safe Radical D3 Right Hemicolectomy for Cancer through Preoperative Biphasic Multi-Detector Computed Tomography (MDCT) Angiography” registered at http://clinicaltrials.gov/ct2/show/NCT01351714

## Introduction

The current guidelines in colorectal cancer treatment^1,2^ use primary tumor (pT), regional lymph node (pN), and distant metastasis in cancer staging for disease stratification. Among these factors, in the current guidelines (AJCC 8th edition), lymph node metastasis is the only important entity for assessing adjuvant treatments.^2^ These guidelines recommend the examination of 12 regional lymph nodes for evaluating the disease. Adjuvant treatment should be considered, even with one positive lymph node. However, these guidelines do not consider the location of the lymph node (D3 or D2 volumes). Although occult tumor cells (OTC) are not mentioned in this stratification,^1,2^ the guidelines state that these “clumps” of 10–20 cells in regional lymph nodes should be treated as positive.^3^ OTC are defined as micrometastases (MM) and isolated tumor cells (ITC). Some studies have shown^4,5^ that the presence of MM may lead to poorer survival rates; however, this is not the case with ITC. Protic et al.^6^ have shown significantly poorer survival rates in node-negative patients with ITC. The last decade has also seen the emergence of new diagnostic tools, such as liquid biopsy and/or circulating tumor cells (CTC),^7,8^ which have been developed for the diagnosis of minimal residual disease (MRD). MRD is described as a small fraction of cancer cells that remain or recur after treatment^9^ and might reflect the tumor burden.^10^ Liquid biopsy/CTC can be useful in postoperative follow-up.^8^ On the other hand, OTC defines the location of the disease, as well as the extent of it,^11^ and has the potential to influence treatment. One-stepped nucleic acid amplification (OSNA) and RT-PCR can be used for upstaging the disease in stage II in up to 25–60% of patients.^12,13^ When the extended D3 mesenterectomy is not performed, the OTC may remain within the patient. The recent introduction of complete mesocolic excision (Hohenberger, 14) has set the quality and extent of surgery in the limelight. Data are emerging suggesting that performing more extensive and patient-tailored surgery has the potential to improve disease-free survival (DFS)^15,16^; this has led to the removal of central lymph nodes. Here, there are some implications that the location of positive lymph nodes according to the level of dissection is of a prognostic value. This may imply that a more extensive mesenterectomy could lead to improved survival rates.^17^

The aim of the current study was to re-stratify patients with stage I–III right-sided colon cancer based on the presence or absence of OTC and/or metastatic lymph nodes in the different levels of surgical dissection and to investigate the impact on oncologic outcomes.

## Materials and Methods

### Dataset

The current study presents analysis of prospectively collected data on a subgroup of consecutive patients included in the ongoing multicenter clinical trial “Safe Radical D3 Right Hemicolectomy for Cancer through Preoperative Biphasic Multidetector Computed Tomography (MDCT) Angiography,” which is registered at http://clinicaltrials.gov/ct2/show/NCT01351714 and ethically approved by the Regional Ethical Committee, South-East Norway (REK Sør-Øst) no. 2010/3354. Patients 18 years or older with potentially curable right-sided colon cancer were included after providing written consent. The included hospitals were Akershus University Hospital (AHUS) (2011–2014), the Vestfold Hospital Trust (VHT) (2011–2014), and Viszeralchirurgie Klinikum Karlsruhe, Germany (KR) (2017–2018).

The modes of access accepted in this clinical trial were laparotomy, laparoscopy,^34^ and robotic access. All the patients were operated on according to the study protocol, and instructions were provided for the initial procedures by 3D reconstruction of vascular anatomy. Operative images after specimen removal were obligatory for quality control at open surgery, while videos were used in laparoscopy/robotic access.

#### Inclusion Criteria

- Patients with histopathological verified adenocarcinoma of the right colon.
- Patients under the age of 75.
- Patients medically cleared by an anesthesiologist for general anesthesia.
- Signed informed consent form.

#### Exclusion Criteria

- Patients with recurrent cancer after previous surgery.
- Patients with distant metastasis.
- Patients not medically cleared to undergo anesthesia.
- Patients who did not sign the informed consent form.

### Surgical Specimen

**Fig. 1:**
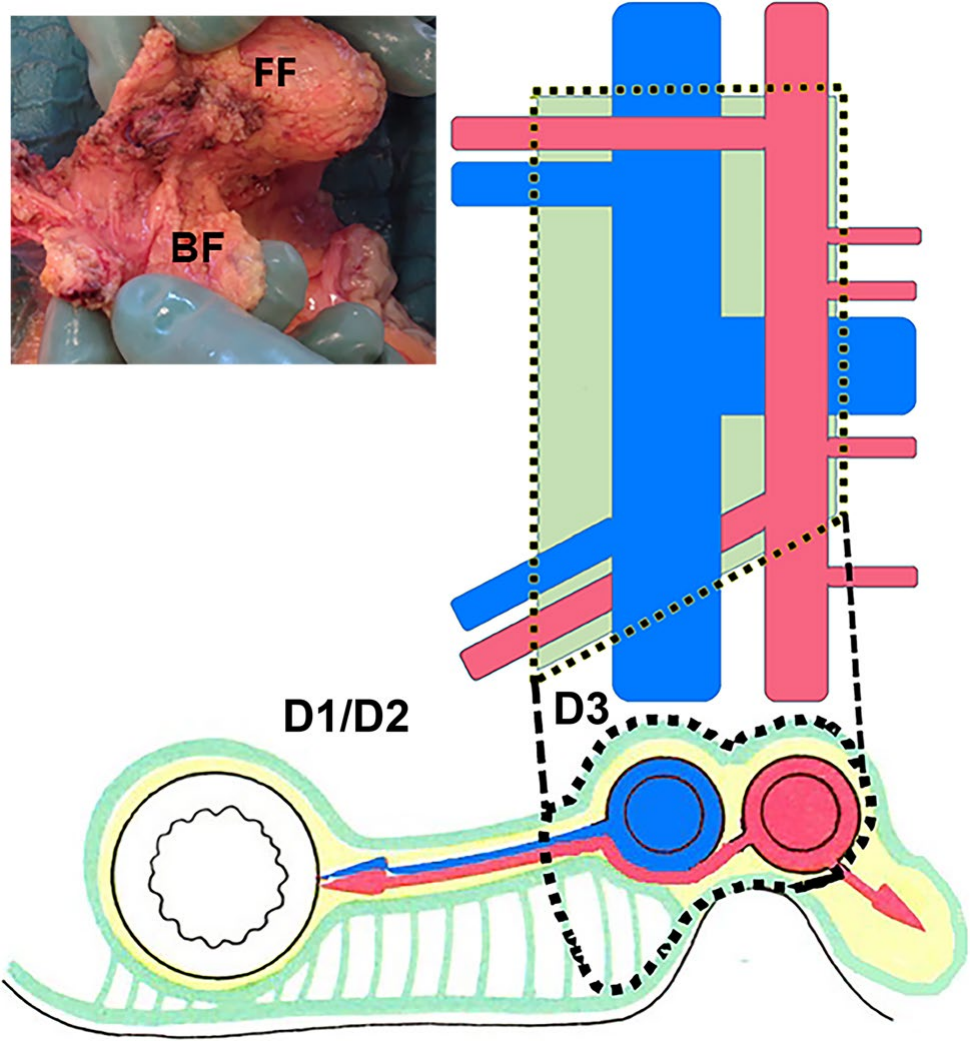
Composition showing D3 area after extended D3 mesenterectomy. FF, front flap; BF, back flap. D1/D2, D1/D2 volume; D3, D3 volume

This current study includes consecutive patients operated only with laparotomy in AHUS and VHT (2011–2014) and KR (2017–2018) from the ongoing trials. The surgical dissection was medial to lateral (devascularization first), with extended mesenterectomy and with the medial limit of dissection along the left border of the superior mesenteric artery, removing all mesenteric lymph nodes ventrally and dorsally to the superior mesenteric vessels^36^ and dissecting the ileocolic pedicle, as well as including complete lymph node dissection along the MCA trunk, with ligation of its right branch or the main trunk. After surgery, the specimen was divided into the respective D1/D2 and D3 volumes through a line 10 mm to the right of the superior mesenteric vein (SMV), 10 mm caudal to the ileocolic artery origin, and 5 mm cranial to the middle colic artery origin,^18,19^ as shown in Fig. 1. The separate D1/D2 and D3 volumes were preserved in a fixative containing glacial acetic acid.^20^

### Histopathology

All histopathological examinations were performed according to the same methodology by Solveig Norheim Andersen (SNA) at AHUS and VHT, while Ulrich Schneider (US) performed the analyses at KR. In specimens from patients with stage I/II disease, all lymph nodes in the D1/D2 and D3 volumes were examined. In specimens from stage III disease, lymph nodes within the D1/D2 volume were not examined for OTC because the disease in this volume was already established. However, all lymph nodes within the D3 volume were assessed for OTC, regardless of whether lymph node metastases had been found.

After routine staining and microscopic evaluation, the lymph nodes in the D1/D2 and D3 volumes were investigated by sectioning into 3–4 µm thin slides before being stained immunohistochemically by using cytokeratin CAM 5.2 antibodies, as stated in a previously published article.^11^ All lymph node sections that were stained immunohistochemically were examined by the same pathologist at the hospital where the patients were included. Both clusters of malignant cells and individual tumor cells were categorized according to the tumor node metastasis (TNM) staging system of the American Joint Committee on Cancer (AJCC):^1,2^ ordinary metastasis: cluster of tumor cells larger than 2 mm in diameter; micrometastasis (MM): malignant cell cluster between 0.2 and 2 mm in diameter; and ITC: tiny cell groups less than 0.2 mm in diameter or single isolated tumor cells (up to 200 cells).

### Adjuvant Chemotherapy

Adjuvant chemotherapy after curative resection was offered to patients under 75 years of age with stage III colon cancer, according to the Norwegian Guidelines for Colorectal Cancer^21^ and the German guidelines of the Program in Oncology.^22^ Patients between 70 and 75 years old received routine monotherapy, either 5-flurouracil (5-FU) or capecitabine (Xeloda). Patients under 70 years of age were routinely offered XELOX in case of N1 stage (6 cycles of capecitabine + oxaliplatin) or XELOX/FOLFOX/FLOX in case of N2 stage (12 cycles of either capecitabine or 5-flurouracil combined with oxaliplatin).

Stages I/II (SS1) or RS1/RS2 were routinely not given adjuvant chemotherapy treatments,^21,22^ with the following exceptions:

- Perioperative tumor perforations.
- If any tumor deposits were found in the histopathological results.

In this substudy, there were no perioperative tumor perforations or upstaging because of tumor deposits in the histopathological findings.

### Grouping of the Patients

**Fig. 2:**
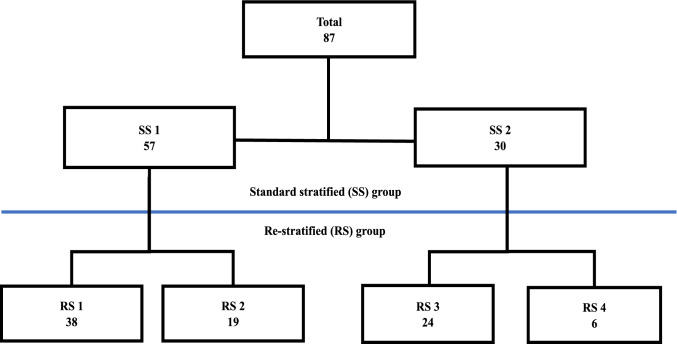
Flow chart standard stratification and re-stratification groups. SS1, stage I (T1-2 N0 M0) and stage II (T3-4 N0 M0). SS2, stage III (any T N1-2 M0). RS1, stage I/II, no OTC in D1/D2 and D3 volumes. RS2, stage I/II, OTC in D1/D2 and/or D3 volumes. RS3, stage III, lymph node metastases in D1/D2, with/without OTC in the D3 volumes. RS4, stage III, lymph node metastases in D3, with/without OTC in D3 volume

All patients were stratified in the following manner (Fig. 2):

1. Standard stratification (SS): The patients were grouped first according to the AJCC classification (8th edition):

SS1: Stage I (T1–2 N0 M0) and stage II (T3–4 N0 M0).

SS2: This includes stage III (any T N1–2 M0).

2. Re-stratification (RS): All patients were re-stratified according to the presence or absence of OTC and lymph node metastases according to their level of dissection (D1/D2 and D3).

Using the abovementioned criteria, the following four patient groups were defined.

RS1: Stages I/II, no OTC in the respective D1/D2 and D3 volumes.

RS2: Stages I/II, OTC in D1/D2 and/or D3 volumes.

RS3: Stage III, patients with lymph node metastases in the level of dissection D1/D2, with or without OTC in the D3 volumes.

RS4: Patients with stage III, lymph node metastases in the level of dissection III (D3 volume), with or without lymph node metastasis at the level of dissection D1 and D2 volume and with or without OTC in the D3 volumes.

### Lymph Node Ratio (LNR) and LNR (OTC)

The LNR ratio was calculated for the SS and RS groups separately. The LNR was calculated by dividing the number of positive lymph nodes by the total number of lymph nodes in the entire specimen, that is, the D1/D2 and D3 levels of dissection. Furthermore, LNR (OTC) was calculated for the RS2 (D1/D2 and D3 volume) and RS4 (D3 volume only) groups.

### Statistical Analysis

The characteristics of the entire sample, as well as stratified by groups SS and RS, were presented as the means and standard deviations (SDs) or frequencies and percentages. The groups were compared by the *χ*^2^ test for categorical variables, while independent samples *t*-test or ANOVA was used for continuous variables. LNR was presented as the mean (SD) stratified by SS and RS. Five-year DFS (5YDFS) and 5-year overall survival (5YOS) were illustrated by the Kaplan–Meier curves and compared by the long-rank test. Results with *p*-values less than 0.05 were considered statistically significant. Statistical Product and Service Solutions Software (SPSS, Inc., Chicago, IL) version 27 for Windows was used for statistical analyses.

## Results

A total of 87 patients (39 men, 44.8%) with a mean age of 68.4 ± 9.9 years were included in this study. The demographic and clinical data for the entire sample and for the SS and RS groups are presented in Table 1. Overall, the 30-day mortality was 5.7% (*n* = 5), all belonging to the stage I/II group. The cause of death was myocardial infarction in two, acute respiratory distress in two, and unknown in one.

**Table 1:** Sample characteristics for all patient groups, standard stratification, and re-stratified by MM/ITC status

Parameters	Total (*N* = 87)	Standard stratification (SS) group			Re-stratification (RS) group				
		Stage I/II (SS1^a^) (*N* = 57)	Stage III (SS2^b^) (*N* = 30)	*p*-value^1^	RS1^6^ (*N* = 38)	RS2^7^ (*N* = 19)	RS3^8^ (*N* = 24)	RS4^9^ (*N* = 6)	*p*-value^5^
Sex, male, *n* (%)	39 (44.8)	24 (42.1)	15 (50.0)	0.482^2^	18 (47.4)	6 (31.6)	13 (54.2)	2 (33.3)	0.455^2^
Age, years, mean (SD)	68.4 (9.9)	68.0 (9.9)	69.2 (9.9)	0.572^3^	69.7 (9.4)	64.5 (10.4)	69.4 (10.2)	68.5 (9.4)	0.273^10^
Male	68.7 (9.6)	68.3 (10.4)	69.5 (8.3)	0.704^3^	69.8 (10.2)	63.7 (10.5)	68.2 (7.9)	78.0 (7.1)	0.287^10^
Female	68.2 (10.2)	67.8 (9.7)	69.0 (11.5)	0.701^3^	69.7 (8.8)	64.9 (10.8)	70.9 (12.6)	63.8 (6.3)	0.353^10^
T stage, *n* (%)									
T1	2 (2.3)	1 (1.8)	1 (3.3)	0.673^2^	1 (2.6)	0	1 (4.2)	0	NA^4^
T2	10 (11.5)	8 (14.0)	2 (6.7)		7 (18.4)	1 (5.3)	2 (8.3)	0	
T3	66 (75.9)	43 (75.4)	23 (76.6)		28 (73.7)	15 (78.9)	18 (75.0)	5 (83.3)	
T4	9 (10.3)	5 (8.8)	4 (13.3)		2 (5.3)	3 (15.8)	3 (12.5)	1 (16.7)	
N stage, *n* (%)				NA^4^					
N0	57 (65.5)	57 (100)	0		38 (100)	19 (100)	0	0	NA^4^
N1	18 (20.7)	0	18 (60.0)		0	0	17 (70.8)	1 (16.7)	
N2	12 (13.8)	0	12 (40.0)	0.166^2^	0	0	7 (29.2)	5 (83.3)	
Tumor differentiation, *n* (%)									
High	18 (20.7)	14 (24.6)	4 (13.3)		9 (23.7)	5 (26.3)	4 (16.7)	0	0.312^2^
Moderate	54 (62.1)	36 (63.1)	18 (60.0)	0.482^3^	25 (65.8)	11 (57.9)	15 (62.5)	3 (50.0)	
Low	15 (17.2)	7 (12.3)	8 (26.7)		4 (10.5)	3 (15.8)	5 (20.8)	3 (50.0)	
Lymph nodes, mean (SD)	40.7 (18.4)	39.7 (16.5)	42.7 (21.8)		41.8 (18.4)	35.6 (11.2)	45.1 (23.1)	32.8 (12.8)	0.260^3^
Location recurrence, *n* (%)				NA^4^					
No metastasis, *n* (%)	71 (81.6)	49 (86.0)	22 (73.3)		35 (92.1)	14 (73.7)	19 (79.2)	3 (50.0)	NA^4^
Liver	5 (5.7)	2 (3.5)	3 (10.0)		0	2 (10.5)	3 (12.5)	0	
Lung	2 (2.3)	1 (1.8)	1 (3.3)		0	1 (5.3)	0	1 (16.7)	
Local	3 (3.4)	0	3 (10.0)		0	0	1 (4.2)	2 (33.3)-	
Other locations	1 (1.1)	1 (1.8)	0		0	1 (5.3)	0	0	
New cancer	5 (5.7)	4 (7.0)	1 (3.3)		3 (7.9)	1 (5.3)	1 (4.2)	0	
Tumor location, *n* (%)				0.303^2^					
Coecum	38 (43.7)	25 (43.9)	13 (43.3)		14 (36.8)	11 (57.9)	10 (41.7)	3 (50.0)	0.214^2^
Ascending	31 (35.6)	21 (36.8)	10 (33.3)		16 (42.1)	5 (26.3)	10 (41.7)	0	
Right flexure	10 (11.5)	6 (10.5)	4 (13.3)		5 (13.2)	1 (5.3)	3 (12.5)	1 (16.7)	
Transversum	6 (6.9)	5 (8.8)	1 (3.3)		3 (7.9)	2 (10.5)	0	1 (16.7)	
Synchronous cancer	2 (2.3)	0	2 (6.7)		0	0	1 (4.2)	1 (16.7)	
Reason of death				NA^4^					
Myocardial infarction	2 (2.3)	2 (3.5)	0		2 (5.3)	0	0	0	NA^4^
Respiratory/COPD	3 (3.4)	3 (5.3)	0		3 (7.9)	0	0	0	
Recurrence	8 (9.2)	2 (3.5)	6 (20.0)		0	2 (10.5)	3 (12.5)	3 (50.0)	
Metachronous cancer	4 (4.6)	3 (5.3)	1 (3.3)		3 (7.9)	0	1 (4.2)	0	
Unknown	2 (2.3)	2 (3.5)	0		2 (5.3)	0	0	0	
Alive	68 (78.2)	45 (78.9)	23 (76.7)		28 (73.7)	17 (89.5)	20 (83.3)	3 (50.0)	
Adjuvant chemotherapy, *n* (%)	23 (26.4)	23 (26.4)	23 (76.6)	NA	0	0	19 (79.7)	4 (66.6)	NA
Recurrence, *n* (%)	7 (8.0)	7 (8.0)	7 (23.3)	NA	0	0	4 (16.6)	3 (50)	NA
5-yr overall survival, *n* (%)	68 (78.2)	68 (78.2)	23 (76.7)	0.875^11^	28 (73.7)	17 (89.5)	20 (83.3)	3 (50.0)	0.144^11^
5-yr disease-free survival, *n* (%)	76 (87.4)	76 (87.4)	23 (76.7)	**0.049**^**11**^	38 (100)	15 (78.9)	20 (83.3)	3 (50.0)	**0.001**^11^

^1^*p*-value comparing stage I/II vs stage III; ^2^*p*-value for *χ*^2^ test; ^3^*p*-value for independent samples *t*-test; ^4^*p*-value not available due to extremely skewed distribution; ^5^*p*-value comparing four groups; ^10^*p*-value for ANOVA; ^11^*p*-value for log-rank test

*SS1*^*a*^, stage I (T1-2 N0 M0) and stage II (T3-4 N0 M0)

*SS2*^*b*^, stage III (any T N1-2 M0)

*RS1*^*6*^, stage I/II, no occult tumor cells (OTC) in D1/D2 and D3 volumes

*RS2*^*7*^, stage I/II, OTC in D1/D2 and/or D3 volumes. Number of OTC:18 isolated tumor cells (ITC) and 1 micrometastasis (MM)

*RS3*^*8*^, stage III, lymph node metastases in D1/D2, with/without OTC in the D3 volumes. One patient had ITC in D3 volumes

*RS4*^*9*^, stage III, lymph node metastases in D3, with/without OTC in D3 volumes. 3 patients had ITC, of which 1 had MM

### Standard Stratification (SS) Group

**Fig. 3:**
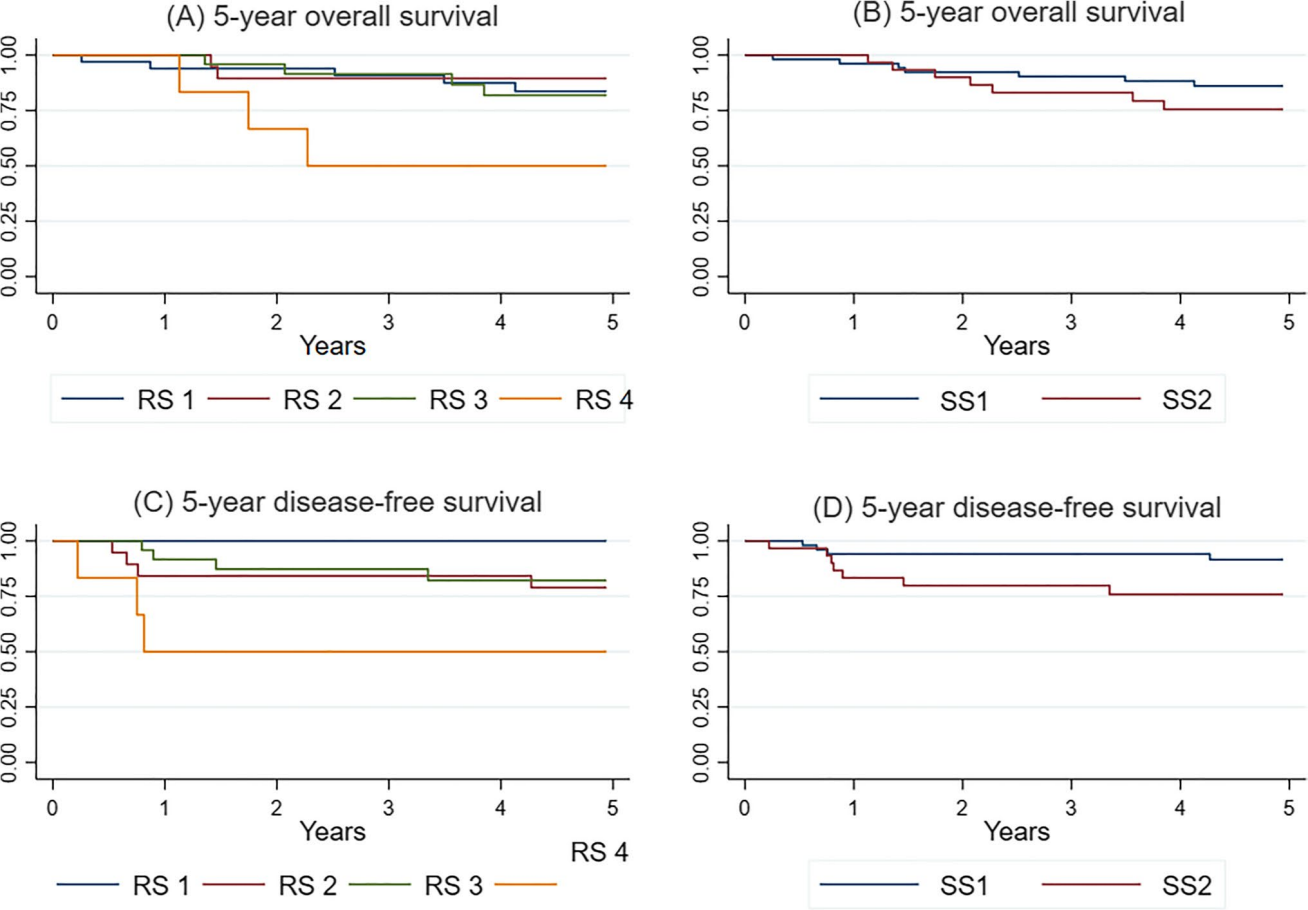
Five-year overall survival and 5-year disease-free survival in re-stratified groups. SS1, stage I (T1-2 N0 M0) and stage II (T3-4 N0 M0). SS2 includes stage III (any T N1-2 M0). RS1, stage I/II, no OTC in D1/D2 and D3 volumes. RS2, stage I/II, OTC in D1/D2 and/or D3. RS3, stage III, lymph node metastases in D1/D2, with/without OTC in the D3. RS4, stage III, lymph node metastases in D3, with/without OTC in D3 volume

SS1 contained 57 patients (24 men, 42.1%), with a mean age of 68.0 ± 9.9. SS2 contained 30 patients (15 men, 50%), with a mean age of 69.2 ± 9.9. The groups were comparable regarding sex, age, tumor differentiation, tumor location, and lymph node harvest (Table 1). There was no significant difference in 5YOS between the groups (*p* = 0.875); however, there was a significant difference in 5YDFS (*p* = 0.049) (Fig. 3).

### Re-stratified (RS) Group

The number of patients per group was 38 in RS1, 19 in RS2, and 24 in RS3, while RS4 contained only six patients. The groups were comparable regarding sex, age, tumor differentiation, tumor location, and lymph node harvest (Table 1). There were no recurrences in RS1, while RS2 and RS3 had four recurrences each. RS4 had three recurrences. Death because of recurrence was 0 (0.0%), 2 (10.5%), 3 (12.5%), and 3 (50.0%) for RS1, RS2, RS3, and RS4, respectively. There was no significant difference in 5YOS between the groups (*p* = 0.144); however, there was a significant difference in 5YDFS (*p* = 0.001) (Fig. 3).

### Lymph Node Ratio (LNR)

The LNR and LNR (OTC) ratio in D1/D2, D3 volume, and complete specimen for SS and RS groups is shown in Table 2. These results show a somewhat higher LNR (OTC) in group RS2 when compared with the LNR of group RS3.

**Table 2:** LNR and LNR (OTC) ratio

	No. of patients	Lymph nodes, mean (SD)	LNR ratio		
			D1/D2 lymph node volume	D3 lymph node volume	Complete specimen
SS groups					
Stage I/II + III	87	40.7 (18.4)	Yes	Yes	0.025 (0.047)
Stage I/II	57	39.7 (16.5)	NA	NA	NA
Stage III	30	42.7 (21.8)	0.141 (0.161)	0.087 (0.246)	0.073 (0.054)
RS groups					
RS1	38	41.8 (18.4)	NA	NA	NA
RS2	19	35.6 (11.2)	NA	NA	NA
LNR (OTC)	19	35.6 (11.2)	0.157 (0.160)	0.035 (0.153)	0.092 (0.081)
RS3	24	45.1 (23.1)	0.113 (0.132)	NA	0.056 (0.026)
RS4	6	32.8 (12.8)	0.253 (0.226)	0.435 (0.411)	0.140 (0.084)
LNR (OTC)	6	32.8 (12.8)	NA	0.390 (0.794)	0.042 (0.062)

*SS1*, stage I (T1-2 N0 M0) and stage II (T3-4 N0 M0)

*SS2*, includes stage III (any T N1-2 M0)

*RS1*, stage I/II, no OTC in D1/D2 and D3 volumes

*RS2*, stage I/II, OTC in D1/D2 and/or D3

*RS3*, stage III, lymph node metastases in D1/D2, with/without OTC in the D3 volumes

*RS4*, stage III, lymph node metastases in D3, with/without OTC in D3 volumes

*LNR ratio*, lymph node ratio

*OTC*, occult tumor cells

### Adjuvant Chemotherapy

A total of 30 (34.5%) of the 87 patients had positive lymph node status (SS2). There were 24 (80%) with metastases in D1/D2 volume (RS3) and 6 (20%) with metastases in the D3 volume (RS4). D3-positive patients constituted 6.9% of the total 87 patients. Patients receiving adjuvant chemotherapy and developing recurrence are presented in Table 1. Seven patients who did not receive adjuvant chemotherapy had a recurrence-free 5-year period (two with positive nodes in the D3 volume).

## Discussion

The most important finding of the current article is that the re-stratification of patients based on the presence or absence of OTC and/or metastatic lymph nodes in the different levels of surgical dissection can identify a patient subgroup with a particularly poor prognosis. A secondary finding is that if the RS4 patient group is removed from the SS2, the remaining patients (RS3) have an identical Kaplan–Meier survival curve as group RS2, which includes those patients who did not routinely receive adjuvant chemotherapy. It is important to note the difference between the survival curves of groups RS2 and RS3 because stage III colon cancer patients routinely receive adjuvant chemotherapy. These results seem to highlight only a delay of recurrence in RS3 patients who have received adjuvant chemotherapy, something that has also been pointed out by Murray et al.^23^ We additionally want to point out that seven stage III patients (23.3%) did not receive adjuvant chemotherapy because of comorbidity and did not develop recurrence (two of these with positive nodes within the D3 volume), implying an effect of extended mesenterectomy.^15,16,24^ This finding is corroborated by Chapuis et al.^25^ that administering chemotherapy to stage III can show a tendency to lower survival rates when compared with stage III patients not receiving chemotherapy.

It also seems that the pattern of microdissemination of the disease follows the same pattern described one century ago.^26^ The overall and disease-free survival curves for stage I/II (SS1) and stage III (SS2) disease do not differ from those previously published.^15,16^ These curves corroborate the current algorithm of adjuvant chemotherapy administration.^21,22^ On the other hand, when, additionally, re-stratifying the patients using OTC, as well as when separating the two different levels of dissection (D1/D2 and D3) into four groups, this is not as obvious anymore, potentially providing an answer to the question posed by Palhman and Hohenberger in their article titled, “Should the Benefit of Adjuvant Chemotherapy in Colon Cancer Be Re-Evaluated?”.^27^

As previously mentioned, after removing stage RS4 patients from the SS2 group, no difference in survival between the RS2 and RS3 groups could be found. Our results also demonstrate that these patients can be readily identified early in the postoperative period, providing that the extended D3 mesenterectomy was performed; this hints at the possibility that only they should be candidates for adjuvant chemotherapy.

When analyzing the treatment options for patients in the re-stratified groups, it seems that the current guidelines correctly address the patients in groups RS1 and RS4. There can be some doubt concerning the patients in the RS2 and RS3 groups. Besides the matching survival curves, RS2 LNR (OTC) and RS3 LNR can imply that these patients represent the same stage of the disease. The AJCC 8th edition states, “It may be better to consider these lymph nodes (containing OTC) as standard positive nodes with the corresponding number, as pathologists likely have considered these to be positive nodes in the past”.^3^

A further factor in this equation is the quality of the surgery performed; this has improved substantially throughout the past decade.^14,15,16,28,29^ The consequence of the improved surgery seems to be a reduction in the number of widespread recurrences, as seen earlier.^30^ It is possible that having a clear definition of the central lymph nodes (D3 volume) allows for the complete removal of these nodes in most patients, thus reducing the incidence of recurrence in them.^17,18,24^ When analyzing the articles on both parallel progression theory^37,38^ (both the primary tumor and lymph node metastases are polyclonal and seeding occurs in multiple waves) or linear progression theory^39,40^ (cancer cells of primary tumors reach the apical lymph node along the adjacent lymph node pathway, leading to distant metastases), a common denominator can be found: radical resection may have a therapeutic effect by removing occult metastatic lymph nodes, implying that tumor biology plays a crucial role in the evolution of the disease. Lal et al.^41^ have recently shown a relationship between the primary tumor immune response and lymph node yield, resulting in improved survival. As seen in our results in the RS3 and RS4 groups, patients with positive nodes in D1/D2 (5/24, 20.8% pts) and D3 (2/6, 33% pts) volumes survived without adjuvant chemotherapy, again implying a significant role of tumor biology.^31,32,33^ According to this, our results seem to support the linear progression theory rather than the parallel progression theory.

The strength of the current article is that it presents an analysis of 87 consecutive patients included in a prospective clinical trial that has a clearly defined volume of mesenterectomy and a clear line of surgical specimen division into the respective level of dissection areas (D2 and D3). The surgery required to achieve such stratification implies the removal of the central lymph nodes (D3 volume) en bloc, representing advanced surgery and dissection within the vascular sheath of the superior mesenteric vessels.^18,34,35^ A possible limitation in this current study can be assessed as being low, especially in group RS4 (only six patients), and can raise a concern about the validity of the results. By all means, a future multi-centric study would fulfill this demand. On the other hand, the SS group survival data according to disease stage demonstrate a completely expected and well-known pattern.

## Conclusion

Re-stratification of patients with stage I–III right-sided colon cancer using OTC presence or absence and/or metastatic lymph nodes in the different levels of surgical dissection has the potential to identify a patient group with poor prognosis, while the remaining patients with stage III disease seem to have the same prognosis as those with OTC in stages I/II of the disease. This may imply in the setting of extended mesenterectomy that the level of positive lymph nodes resected may have a significant impact on disease-free survival.

## Data Availability

Available on request.
